# The Possible Role of Plant-Based Bars Consumption in CKD Geriatric Patients

**DOI:** 10.3390/ph17121689

**Published:** 2024-12-14

**Authors:** Giulia Marrone, Silvia Urciuoli, Manuela Di Lauro, Kevin Cornali, Claudia Masci, Manfredi Tesauro, Pamela Vignolini, Annalisa Noce

**Affiliations:** 1 Department of Systems Medicine, University of Rome Tor Vergata, 00133 Rome, Italycornali.kevin@hotmail.it (K.C.);; 2 Department of Statistics, Computer Science and Application—PHYTOLAB (Pharmaceutical, Cosmetic, Food Supplement, Technology and Analysis), University of Florence, Sesto Fiorentino, 50019 Florence, Italy; silvia.urciuoli@unifi.it (S.U.); pamela.vignolini@unifi.it (P.V.); 3 UOSD Nephrology and Dialysis, Policlinico Tor Vergata, 00133 Rome, Italy

**Keywords:** *Crocus sativus* L., elderly, chronic kidney disease, antioxidants, sarcopenia, depressive disorders, *Olea europaea* L., *Vitis vinifera* L., polyphenols

## Abstract

**Background**: Chronic kidney disease (CKD) geriatric patients experience a premature aging process, compared with the general population of the same age and sex. The uremic milieu is capable of enhancing oxidative stress (OS) and microinflammation, leading to a pro-aging mechanism and an increased protein catabolism. Moreover, cognitive disorders are observed. Objectives: The aim of this pilot study was to evaluate the possible beneficial effects on the body composition, cognitive functions, inflammatory state and OS of CKD–geriatric patients induced by the consumption of two different plant-based bars (PBBs). **Methods**: A total of 20 male (mean age 73 ± 7 years) and 9 female patients (mean age 71 ± 4 years) were enrolled, divided as follows: 19 in the PBBs group (that consumed both bars) and 10 in the control group. They were monitored for 12 weeks. The PBBs presented a moderate caloric value and were enriched with waste and by-products of wine and olive oil supply chains and with organic saffron. **Results and Conclusions**: At the end of this study, the PBBs group, compared to the control group, showed an improvement in their body composition, detected by bioimpedance analysis and ultrasound examination, and in their cognitive function, revealed by mini-mental state examination. In the PBBs group, we also observed an OS reduction, through the free oxygen radical test.

## 1. Introduction

Old age is defined as the final stage of the aging process, leading to a progressive decline of body functions until death. In Europe, the World Health Organization (WHO) has adopted a threshold of 60 years of age to define geriatric age. It generated a new classification of “old patients”, divided into three sub-groups: “young-old patients” (60–74 years old), “old-old patients” (75–90 years old) and “oldest-old patients” (>90 years old) [1]. This is mainly due to the increase in life expectancy of the general population, which is posing new challenges to healthcare systems worldwide, as are the costs arising from the treatment of chronic degenerative non-communicable diseases (CDNCDs) [2]. Among the main CDNCDs, chronic kidney disease (CKD) plays a central role as it is a very common manifestation in elderly patients [3]. In the general population, the process of human aging is complex, irreversible, individualized and it involves biological, psychological and social factors. Biological factors include physical inactivity, malnutrition and acute and chronic diseases. These factors are able to alter cell metabolism and the self-regulation and the regeneration of tissues and organs. These changes result in cell atrophy, in internal and external cell dehydration, in the increase of fat mass, in the reduction of muscle mass (MM) and in the deposition of amyloid-ß plaques in the brain. The psycho-social factors are related to biological changes. In fact, the latter affect the humor, physical condition and social activity of the elderly, leading them to isolation, loneliness and incapacity in facing old age. Moreover, during aging, short-term memory, visual–spatial memory and the ability to represent mental processes tend to deteriorate [4]. However, in this complex puzzle, some CDNCDs, such as CKD itself, induce a premature and critical aging process. In CKD patients, the uremic milieu is capable of enhancing oxidative stress and microinflammation, thus provoking the activation of a stress-resistance response. This contributes to the inhibition of anabolic pathways and of anti-aging mechanisms, leading to an increased protein catabolism and pro-aging mechanism. These factors, in combination with alterations in the sympathetic–vagal balance and in circadian rhythm, constitute a premature aging phenotype in CKD patients. This uremic phenotype is characterized by osteoporosis, frailty, cardiac hypertrophy, vascular calcifications, muscle wasting and protein-energy wasting (PEW) syndrome [5,6]. PEW syndrome is a term proposed by the International Society of Renal Nutrition and Metabolism to describe a state of nutritional and metabolic derangements in CKD patients, characterized by the simultaneous loss of systemic body proteins and energy stores. During PEW, increased protein catabolism and anabolic resistance mask muscle protein synthesis to anabolic stimuli. The resulting alterations in body composition are responsible for the worsening of renal function, health-related quality of life (QoL), depression and the presence of malnutrition, which can lead to an increased risk of frailty, hospitalization and mortality [7]. Microinflammation is commonly observed in CKD patients. In fact, both the progressive loss of kidney function and old age are related to an increased production of inflammation markers, such as C-reactive protein (CRP) and fibrinogen, and an enhanced release of pro-inflammatory mediators, such as interleukin (IL)-6, tumor necrosis factor (TNF)-α and IL-1β [8]. This inflammatory status, associated with oxidative stress (OS), is one of the main mechanisms involved in PEW syndrome. In CKD patients, this comorbidity is recognized as a diagnostic criterion of an inflammatory state, manifesting as a reduction in serum albumin. The other diagnostic criteria are body weight loss, body fat loss, MM loss and reduction in energy and/or in protein intake. In addition to pharmacological therapy, nutritional therapy is also fundamental in delaying signs and uremic symptoms associated with the disease itself [9]. In our previous studies, we demonstrated how an innovative nutritional therapy, based on the consumption of functional foods (FFs), formulated ad hoc for CKD patients, was able to counteract several CKD-related comorbidities, such as microinflammation, dyslipidemia and uremic sarcopenia and to improve CKD patients’ QoL. These FFs were in the form of bars, which were obtained via a circular economy model to recover a high amount of natural bioactive compounds (NBCs) from different agro-food chains, and were administered for three months, twice a day [10]. Fruit and vegetables are the richest source of food phenolic compounds. A high consumption of these foods has been linked to a reduction in the risk of the most common CDNCDs, caused by oxidative stress [11], but the antioxidant prevention of polyphenols against CKD has been poorly studied.

In this context, we decided to evaluate how, in CKD geriatric patients, the consumption of organic plant-based bars, based on innovative and functional ingredients coming from the circular recovery of waste and by-products of wine and olive oil supply chains and containing organic saffron, may be able to counteract PEW syndrome and to improve the cognitive functions and the QoL of these patients.

Two PBBs were chosen and provided free of charge by FunctionX srl (Latina, Italy). The peculiarity of these bars was the presence of innovative and functional ingredients, coming from the circular recovery of waste and by-products of wine and extra-virgin olive oil (EVOO) supply chains, and of organic saffron. In particular, the bars contained micronized grape pomace, grape seeds and olive leaves from sustainable and circular processes, that enable the enhancement of the use of waste and by-products rich in polyphenolic bioactive compounds [12].

The transformation of wine and EVOO supply chain by-products into secondary raw materials can improve the efficiency and the sustainability of these sectors. Therefore, it opens up new opportunities for agrifood wastes business, innovation and valorization. This approach protects the environment and responds to the growing demand for more sustainable food products.

## 2. Results

The results of routine blood and urine tests, monitored at two time points during this study, in the plant-based bars (PBBs) group and in the control group were not statistically significant.

The results of the measurement of oxidative stress and of inflammatory parameters, analyzed in the PBBs group and in the control group, are reported in Table 1. At the end of this study, a reduction in free oxygen radical test (FORT) levels (*p* = 0.0374) in the PBBs group compared to the control group was observed.

The results of body composition analysis, performed using bioimpedence analysis (BIA), for the PBBs group and the control group are reported in Table 2. At the end of this study, we observed a reduction in resistance (*p* = 0.0166) in the PBBs group compared to the control group. Moreover, we observed a significant increase in fat free mass (FFM) (*p* = 0.0226) and body cell mass index (BCMI) (*p* = 0.0313), with a concomitant significant decrease in extracellular water (ECW) (*p* = 0.0476) and fat mass (FM) (*p* = 0.0182) in the PBBs group compared to the control group. Finally, we detected a significant increase in quadriceps rectus femoris thickness (QRFT), both on the right and left, at all landmarks, in the PBBs group compared to the control group.

The following tables (Table 3 and Table 4) show the results of the handgrip strength test (HGST) and of systolic and diastolic blood pressure (BP), examined in the PBBs group and in the control group, at the two time points during this study. These results do not show statistically significant variations.

**Figure 1:**
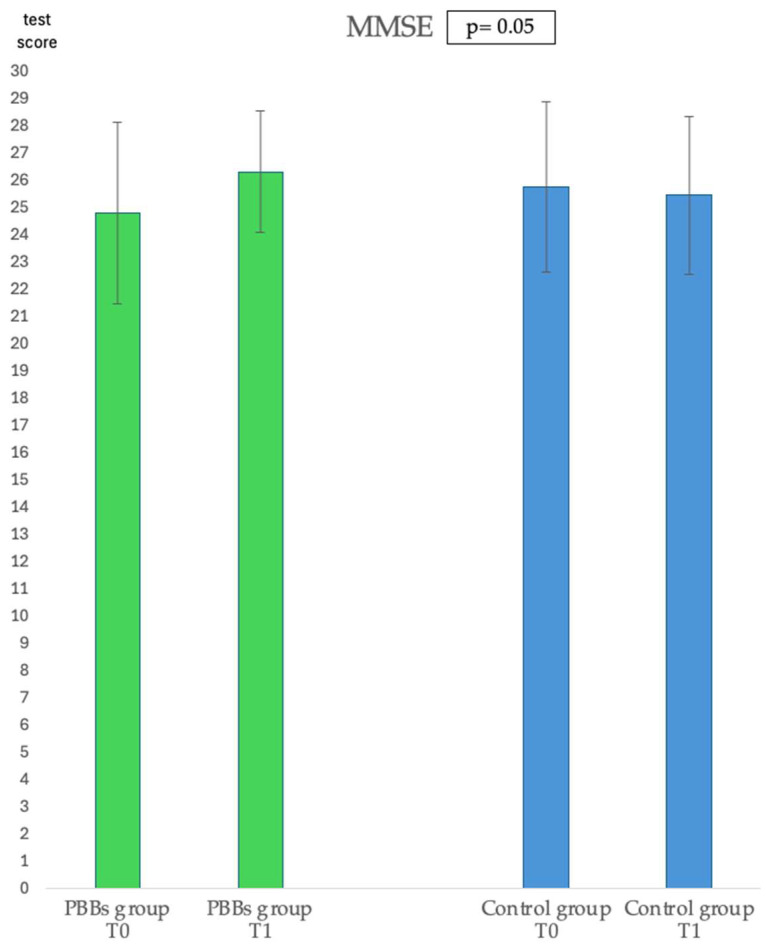
Results of the mini-mental state examination (MMSE) administered in the plant-based bars (PBBs) group and in the control group at the two time points during this study.

The mini-mental state examination (MMSE) represents a rapid and sensitive tool for the exploration of cognitive function and its changes over time. Regarding the MMSE questionnaires administered, at the two time points during this study, we observed a statistically significant improvement in the PBBs group (*p* = 0.05) at the end of the study compared to the control group (Figure 1). The 36-Item Short-Form Health Survey (SF-36), Baecke, Prevención con Dieta Mediterránea (PREDIMED) and International Physical Activity Questionnaire (IPAQ) questionnaires revealed no statistically significant differences in both the control group and in the PBBs group at the two time points during this study.

## 3. Discussion

The alteration of nutritional status and body composition is common in CKD patients. In particular, in advanced CDK stages, we observed PEW syndrome. One of the main factors underlying the etiopathogenesis of PEW is reduced calorie and protein intake compared to that recommended by international guidelines [13,14]. This phenomenon is related to poor appetite, uremic gastritis, depression, the hypercatabolic state and the numerous dietary restrictions that characterize CKD nutritional therapy [15,16,17]. Inadequate calorie as well as protein intake is associated with a significant decrease in nutritional biomarkers (such as hypoalbuminemia) and a rapid progression of CKD vs. end-stage-kidney disease [18].

In this context, one of the main strategies to counteract the onset of malnutrition is to ensure an adequate caloric intake, i.e., 25–35 kcal/kg of body weight per day, for the patient, to be adjusted according to age, level of physical activity and body composition [14,19].

In our study, we obtained numerous beneficial effects in the parameters related to body composition in patients belonging to the PBBs group compared to the control group. In particular, we observed an increase in FFM% and BCMI, with a concomitant significant decrease in ECW% and FM%. Furthermore, we also observed an improvement in the QRFT of both limbs. These beneficial effects are likely to be attributed to the additional caloric intake provided by the two PBBs, which provided a caloric surplus of 254 kcal per day, compared to the patient’s usual diet. The main source of calories of the two PBBs is attributable to their content of cocoa butter and EVOO, notoriously high-calorie foods [20]. This caloric surplus allowed patients to reach the caloric intake recommended by the guidelines, often slightly increased compared to those of the general population of equal age, because CKD patients are characterized by a microinflammatory state, enhanced oxidative stress and metabolic acidosis that induce hypercatabolism.

Fruits and vegetables are sources of phenolic compounds, vitamins, minerals and phytochemicals with antioxidant action and delay the aging process. Many natural antioxidants are highly effective in controlling oxidative damage generated by free radicals by reducing them [21].

The PBBs used for this study were based on fruits and vegetables partially coming from the circular recovery of waste and by-products from the Italian wine (grape pomace and grape seeds) and EVOO (olive leaf) supply chains and containing only Italian organic EVOO, as a major source of fat, and organic saffron.

The choice of micronized grape seeds and grape pomace as functional and sustainable ingredients is due to the multiple positive effects on the health of geriatric patients. Motohashi et al. analyzed the effects of quercetin and proanthocyanidins, which have immunomodulatory effects in inflammatory conditions with a consequent production of nitric oxide and prostaglandins. These molecules exert anti-inflammatory action by removing free radicals, preventing lipid peroxidation and inhibiting the formation of proinflammatory cytokines [22]. The idea of adding saffron to the PBBs originated from the countless studies about the benefits it exerts, especially in the elderly. Saffron was chosen since many studies have evaluated the effect of this spice and of its bioactive compounds in several pathological conditions. In particular, its potential neuroprotective role in neurodegenerative diseases (e.s. Alzheimer’s, Parkinson’s and Huntington’s diseases) has been examined [23]. Even in our previous review, we clarified how all the components of *Crocus sativus* L. are able to exert countless beneficial actions in various pathologies, such as degenerative maculopathy, depression and anxiety, neurodegenerative diseases, CKD, etc. [24]. Furthermore, saffron appears to be generally well tolerated without any major adverse effects associated with its daily consumption [25].

The daily intake of fruit and vegetables contributes to enhance the antioxidant defenses of the body. In fact, the long-term consumption of foods rich in polyphenols offers protection against the development and the progression of various CDNCDs. In our study, the reduction of FORT levels in the PBBs group confirmed the antioxidant action of the polyphenols, which, thanks to their chemical structure, act as scavengers of free radicals and reactive oxygen species (ROS) [26].

Regarding the cognitive aspect, there are numerous studies that link the consumption of polyphenols from fruit and vegetables and cognitive improvements. Letenneur et al. in 2007 demonstrated a positive association between the intake of foods rich in flavonoids (quercetin, kaempferol and luteolin) and better cognitive evolution thanks to a 10-year study [27]. In 2021, Yang at al. published a review about the potential neuroprotective effect of polyphenols which showed a significant association between the intake of food with high polyphenol content and better cognitive performance [28].

The presence of saffron in the bars may have contributed to cognitive improvements in the PBBs group. Saffron is a source of many bioactive constituents with potential health benefits. One of the most studied effects of saffron is its protective action against some neurocognitive pathologies, such as anxiety, depression, Alzheimer’s disease, attention deficit and impairment of cognitive function [28]. Crocin present in saffron may have a preventive and/or therapeutic role in neuroprotection, including dementia, considering its antioxidant and anti-inflammatory properties. Saffron can be defined as a multifunctional natural product for the brain [29].

Furthermore Ayati et al. highlighted the potential role of saffron in improving cognitive functions and the daily routines of patients with Alzheimer’s disease and mild cognitive impairment [30].

## 4. Materials and Methods

**Figure 2:**
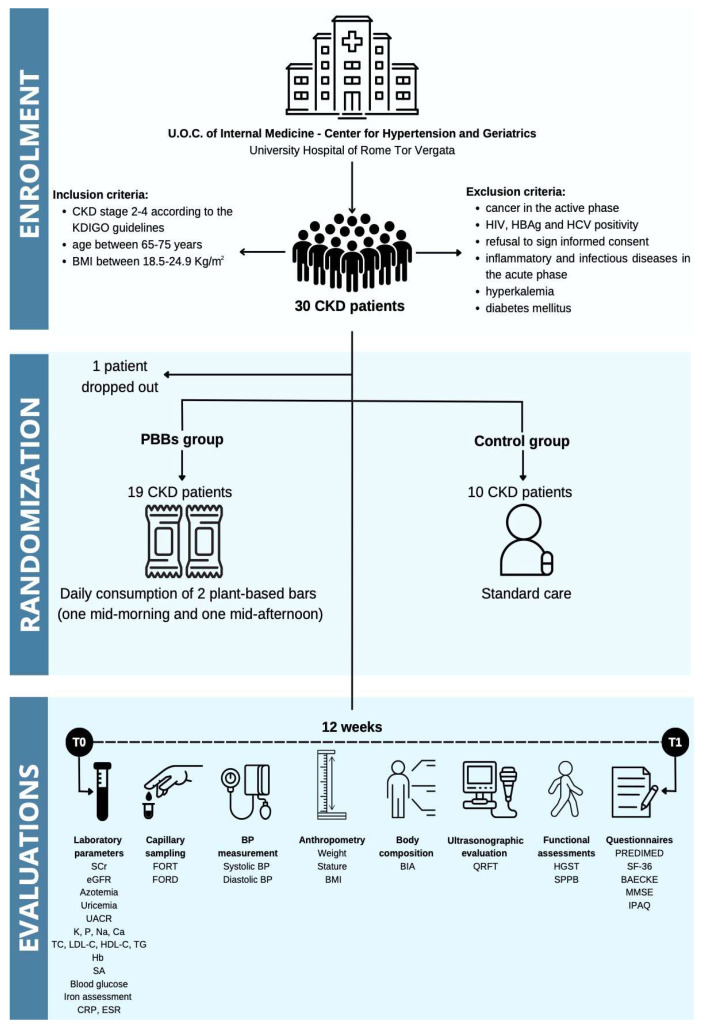
Flow-chart of the study. Abbreviations: BIA, bioimpedance analysis; BMI, body mass index; BP, blood pressure; Ca, calcium; CKD, chronic kidney disease; CRP, C-reactive protein; eGFR, estimated glomerular filtration rate; ESR, erythrocyte sedimentation rate; PBBs, plant-based bars; FORD, free oxygen radical defense; FORT, free oxygen radical test; Hb, hemoglobin; HBsAg, hepatitis B surface antigen; HCV, hepatitis C virus; HDL-C, high-density lipoprotein cholesterol; HGST, handgrip strength test; HIV, human immunodeficiency virus; IPAQ, international physical activity questionnaire; K, potassium; KDIGO, kidney disease: improving global outcomes; LDL-C, low-density lipoprotein cholesterol; MMSE, mini-mental state examination; Na, sodium; P, phosphorous; PREDIMED, prevención con dieta Mediterránea; QRFT, quadricep rectus femoris thickness; SA, serum albumin; SCr, serum creatinine; SF-36, 36-Item Short-Form Health Survey; SPPB, short physical performance battery; TC, total cholesterol; TG, triglycerides; UACR, urinary albumin-to-creatinine ratio; U.O.C., Complex Operating Unit.

The trial population consisted of 30 CKD patients under conservative therapy (stage 2–4, according to the Kidney Disease: Improving Global Outcomes—KDIGO guidelines) [31], who were enrolled at the U.O.C. of Internal Medicine—Center for Hypertension and Geriatrics of the University Hospital of Rome Tor Vergata. Before the division of the groups, one patient dropped out from the study; therefore, the final number of patients was 29—20 males (mean age 73 ± 7 years) and 9 females (71 ± 4 years). Inclusion and exclusion criteria are reported in Figure 2. The experimental protocol complied with the 1975 guidelines of the Declaration of Helsinki and was approved by the Independent Ethical Committee of Policlinico Tor Vergata. The study population was divided into 2 subgroups, homogeneous in terms of age, gender and body mass index (BMI). In particular, 19 patients belonged to the PBBs group, while 10 patients belonged to the control group. The first group consumed two PBBs (named the Green bar and Blue bar) daily, one mid-morning and one mid-afternoon, while the second group followed the usual care. All patients were evaluated at the time of the enrollment (T0) and after twelve weeks (T1) through various assessments shown in Figure 2.

### 4.1. Laboratory Parameters and Capillary Sampling

The laboratory parameters and the capillary sampling, performed at the two study time points, are shown in the Figure 1. All laboratory parameters were analyzed using a Dimension Vista 1500 (Siemens Healthcare Diagnostics, Milano, Italy), except for the lipid profile, which was determined using standard enzymatic colorimetric techniques (Roche Modular P800, Roche Diagnostics, Indianapolis, IN, USA). All parameters were analyzed according to standard procedures in the clinical chemistry laboratories of the University Hospital of Rome Tor Vergata. The capillary sampling served for the execution of the FORT and free oxygen radical defense (FORD) tests, which were analyzed using a CR4000 photometer (Callegari 1930, Parma, Italy). This method, through various solution-catalyzed colorimetric reactions, is able to determine the concentration of reactive oxygen species (FORT) and antioxidant compounds (FORD test). One FORT unit corresponds to 0.26 mg/L hydrogen peroxide (H_2_O_2_). FORT values below 300 U were considered “optimal”, between 300 and 330 U “borderline” and above 330 U indicated the presence of oxidative stress; FORD test values above 1.53 mmol/L Trolox equivalents were considered “optimal”, between 1.53 and 1.07 mmol/L Trolox equivalents “borderline” and below 1.07 mmol/L Trolox equivalents “low” [31].

### 4.2. Questionnaires

At both time points during this study, T0 and T1 (after 12 weeks), five questionnaires were administered to all patients:

- The PREDIMED questionnaire, used to exclude possible biases due to changes in patients’ eating habits. This questionnaire comprises 14 items, each corresponding to 1 point, up to a maximum of 14 points. According to the scoring, patients were divided into three Mediterranean diet adherence groups: minimal (≤5 points), medium (between 6 and 9 points) and maximum (≥10 points) [32];
- The SF-36 questionnaire, used to assess the health-related QoL of patients. It consists of 36 questions divided into nine spheres: the perception of general health (5 questions), health changes (1 question), physical functioning (10 questions), perception of pain (2 questions), social functioning (2 questions), emotional well-being (5 questions), fatigue (4 questions), activity limitations due to health status (4 questions) and activity limitations due to emotional state (3 questions). For each sphere, it is possible to obtain values between 0 and 100, which directly correlate with the patient’s psychophysical well-being [33];
- The Baecke questionnaire, used to assess the physical activity level of patients. This questionnaire examines three domains: work, sports and leisure activities. The following formulas were used to calculate the score for each domain [34]:
  - work index = ((6 − (points for sitting)) + (sum of the points for the other 7 parameters))/8;
  - sport index = (sum of the points for all 4 parameters)/4;
  - leisure index = ((6 − (points for television watching)) + (sum of the points for the remaining 3 items))/4.
- The MMSE, used to assess the cognitive function of patients. The MMSE investigates five different domains: orientation, memory, attention and calculation, recall of three words, and language, for a total of 30 points. A higher score indicates better cognitive function. Mild cognitive impairment (MCI) is identified using specific cut-off points that consider the patient’s age and education level. For patients over 65 years of age, a score of less than 24 points is generally indicative of a significant MCI; for patients over 80 years of age, it is considered at a cut-off of 22 points, since a slight decline in cognition may be attributable to age rather than to a real cognitive impairment [35]. Regarding the level of education, MMSE ≤ 19 points indicates illiterate individuals, ≤22 points indicates participants with primary school education and ≤26 points indicates those with middle school education and above [36]. For CKD patients, no specific cut-offs have been developed but it has been observed that decline of renal function is directly correlated with reduction in MMSE score [37].
- The IPAQ, used to evaluate the grade of physical activity before and after the treatment with PBBs, in order to exclude possible changes in laboratory and body composition parameters induced by a different level of physical activity. The questions aim to define how long an individual has been engaged in intense, moderate and sitting physical activities within the last 7 days. For the three physical activity spheres, the respective metabolic equivalent of tasks (Mets) are calculated as follows:
  - Mets for intense physical activities: 8 Mets × time (minutes) × number of days;
  - Mets for moderate physical activities: 4 Mets × time (minutes) × number of days;
  - Mets for walking activities: time (minutes) × number of days × 3, if moderate; ×3.3, if intense; or ×2.5, if slow.

Total Mets = Mets for intense physical activities + Mets for moderate physical activities + Mets for walking activities.

The grade of physical activity is defined as follows [38]:

- Mets < 700: inactive individual;
- 700 < Mets ≤ 2519: sufficiently active individual;
- Mets > 2520: active or very active individual.

### 4.3. Measurement of Anthropometric Parameters and Body Composition Assessment

Body weight (kg) and stature (m) of the enrolled patients were collected by using a Seca scale (model 700, Hamburg, Germany) with a built-in stadiometer. The measurements were made to the nearest 0.01 kg for body weight and 0.1 cm for stature. The BMI of the patients was calculated by dividing the body weight by stature squared (kg/m^2^). The BIA was performed using an EGF Plus^®^, based on bioelectrical impedance vector analysis (BIVA) technology, at 50 kHz frequency. The software Bodygram HBO 1.1.1.31 (Estor, Pero, MI, Italy) was used to assess patients’ body composition and the following parameters were recorded: resistance (Rz, Ω), reactance (Xc, Ω), phase angle (°), TBW (%), ECW (%), FM (%), FFM (%) and BCM (%). For the execution of the BIA, we used the standardized procedures.

### 4.4. Blood Pressure Monitoring

Systolic and diastolic BP and heart rate were monitored at the two study time points. The measurements were recorded by an automatic sphygmomanometer: the arm cuff was located at the heart level and the third BP measurement was recorded [39].

### 4.5. Ultrasonographic Evaluation

To detect the loss or the improvement of MM in CKD patients, at T0 and T1, all patients underwent an ultrasound examination of the QRFT at ^1^/_2_ (the midpoint between the antero-superior iliac spine and the upper limit of the patella) and at ^2^/_3_ (the boundary point between the lower third and the upper two-thirds of the quadriceps muscle). This ultrasound technique is considered an innovative diagnostic tool capable of reflecting the patient’s total MM. The ultrasonographic evaluation was carried out by the same operator (A.N.) with ultrasound equipment Esaote MyLab70 XVision (Genova, Italy) and a linear probe LA523, through B-mode modulation with a 7.5 MHz transducer. In order to perform the ultrasonographic evaluation, three measurements were recorded bilaterally for both muscle anatomical landmarks, in the supine position and with both knees in extension. The mean value of the three measurements was reported. The probe was placed perpendicular to the long axis of the muscle, covered with an abundant gel layer, on which minimal external pressure was exerted in order to prevent its compression [40,41].

### 4.6. Evaluation of Muscle Strength and Physical Performance

Functional assessments were carried out at baseline (T0) and after 12 weeks (T1):

- Muscle strength was evaluated through the HGST, a dynamometer that assesses handgrip force (Jamar Plus, Performance Health (Warrenville, IL, USA)). The seated patients were asked to squeeze the dynamometer as hard as possible with the elbow of the working hand at 90° close to the hip. The test was performed three times with both limbs alternately and the average value was considered. The pathological cut-offs of HGST are <30 kg for men and <20 kg for women [42].
- Physical performance was evaluated through the short physical performance battery (SPPB), a battery of exercises including gait speed (4 m walking), power (five-times chair sit to stand) and balance (tandem test). Each test is scored up to 4 points and their sum indicates the level of performance. A sum of 12 points is the best score [43].

### 4.7. Composition of the Plant-Based Bars

For this study, two organic PBBs were chosen and provided free of charge by FunctionX srl (Latina, Italy). These bars were designed by the company in collaboration with researchers from the University of Florence.

As reported in Table 5, the two PBBs are based on fruit and vegetables, containing Italian EVOO as a major source of fat. In Table 6, the nutritional information such as fats, carbohydrates, fiber, proteins and salt per 100 g of the two PBBs is reported. The peculiarity of these bars is the presence of innovative and functional ingredients coming from the circular recovery of waste and by-products of the wine and olive oil supply chains. In particular, the Green and Blue bars contain micronized grape pomace, grape seeds and olive leaves from sustainable and circular processes, that enable and enhance the use of waste and by-products rich in polyphenolic bioactive compounds [12].

The active molecules present in the innovative ingredients (secondary raw materials), obtained from circular and sustainable processes, come from Italian olive trees and grapes. The *Olea europaea* L. bioactive molecules are: hydroxytyrosol, tyrosol, oleuropein, verbascoside, oleocanthal and oleacein [44]. *Vitis vinifera* L. is characterized by the presence of cyanidin, malvidin, peonidin and quercetin and its derivatives [45]. Saffron was also added to the Green bar, as it is a functional ingredient for its multiple biological activities [24].

Total antioxidant capacity (TAC) was evaluated using the Folin–Ciocanteu method and the percentage antiradical activity (AA%) was evaluated using the 2,2-diphenyl-1-picryl-hydrazyl-hydrate (DPPH) radical assay as reported by Luzi et al., 2021 [46]. The Green bar had a TAC of 164.9 mg of gallic acid (GAE)/bar and AA of 82.8%. The Blue bar had a TAC of 205 mgGAE/bar and an AA of 85.7%. FunctionX srl provided the energy content data (kcal): for the Green bar, this was 125 kcal/bar and for the blue bar this was 129 kcal/bar. Both bars weighed 32 g.

### 4.8. Statistical Analysis

All data were entered into an Excel spreadsheet (Microsoft, Redmond, WA, USA) and analysis was performed using the Windows Social Science Statistics Package, version 25.0 (IBM_SPSS, Chicago, IL, USA). The data are reported as mean ± standard deviation for the parameters with normal distribution (after confirmation with histograms and the Kolmogorov–Smirnov test), and as medians and intervals (min; max) for variables with non-normal distribution. The one-way ANOVA test was conducted to verify the homogeneity of the epidemiological–anthropometric data among the patients enrolled in this study.

For statistical analysis, an inferential analysis was performed using parametric tests (Student’s t, Fisher’s F, etc.) for quantitative quantities, and non-parametric tests (Mann–Whitney, Fisher’s exact test, etc.) for qualitative variables. The non-parametric median test was used to analyze categorical data.

The results were obtained by comparing the differences (at the two time points) between the study groups. Tests with *p*-values ≤ 0.05 were considered statistically significant.

## 5. Conclusions

Considering the results of this study and the previous scientific literature, we can state that it is possible to use secondary raw materials from the recovery of waste and by-products of the Italian wine and EVOO supply chains as innovative ingredients for the formulation of functional foods with high antioxidant activity [12]. This allowed us to valorize waste by applying the principles of the circular economy to two of the most important Made in Italy supply chains. This study could pave the way for further applications in the nutraceutical sector of innovative circular ingredients rich in antioxidant bioactive compounds.

PBBs, in CKD–geriatric patients, seems to be able to improve body composition, in particular muscle mass, cognitive function and OS.

Therefore, these functional foods could represent a new tool in the clinical management of these patients in order to support traditional pharmacological treatments. In recent years, it has become very important to implement new preventive strategies able to counteract the onset and to slow-down the progression of age-related comorbidities (such as PEW, caused not only by aging but also by CKD itself) in order to improve the QoL of the elderly population. As our promising data were collected only from 29 patients, they need to be confirmed by a larger study population with a longer follow-up period. Moreover, it would be very interesting to analyze the impact of PBBs on other biomarkers, such as inflammatory cytokines.

## Figures and Tables

**Table 1:** Laboratory parameters of the plant-based bars (PBBs) group and of the control group examined at the two time points during this study.

	PBBs Group		Control Group		*p*-Value
Parameter	T0	T1	T0	T1	
FORT (U)	329.5 (196–600)	262.5.5 (160–538)	338 (246–340)	358 (233–346)	0.0374
FORD (mmol/L)	0.9 (1.25–3.14)	1.42 (1.18–2.33)	0.62 (0.63–1.21)	1.2 (0.73–1.3)	ns
CRP (mg/L)	1.2 (0.78–5.6)	1.0 (0.9–30)	5.4 (0.5–5.8)	4.4 (0.5–4.6)	ns
ESR (mm/h)	31 (3–92)	32 (2–83)	92 (43–96)	85 (48–88)	ns

The data are reported as medians (minimum–maximum). Abbreviations: CRP, C-reactive protein; ESR, erythrocyte sedimentation rate; FORD, free oxygen radical defense; FORT, free oxygen radical test; n.s., not significant; T0, enrollment time; T1, after twelve weeks.

**Table 2:** Body composition parameters of the plant-based bars (PBBs) group and of the control group examined at the two time points during this study.

	PBBs Group		Control Group		*p*-Value
Parameter	T0	T1	T0	T1	
Weight (kg)	72 (38.5–91.5)	72 (38–91.5)	71.5 (63.6–74.5)	73.5 (62.5–75)	ns
BMI (kg/m^2^)	26.8 (18.1–33.1)	26.9 (17.8–33.1)	28.7 (23.6–29.7)	27.8 (23.2–29.8)	ns
Resistance (Ω)	502 (430–665)	482 (424–599)	490 (488–740)	494 (494–694)	**0.0166**
Reactance (Ω)	49 (38.7–60)	47 (33–72)	46 (44–48)	48 (43–47)	ns
Phase angle (°)	5.1 (4.4–6)	5.7 (4.3–7.3)	5.1 (3.7–5.2)	5.3 (3.8–5.4)	ns
TBW (%)	54.5 (45–62.5)	55.5 (47.2–68.6)	44.9 (45.6–48.9)	47.3 (47.9–48.3)	ns
ECW (%)	50.3 (45.6–54.2)	47.1 (40.5–55)	50.9 (49.9–59.3)	49.4 (49–58.7)	**0.0476**
FM (%)	26.1 (14.3–39.1)	24.45 (5.9–35.5)	35.8 (33.8–38.1)	33.5 (34.5–35.1)	**0.0182**
FFM (%)	73.9 (60.9–85.7)	75.6 (64.5–94.1)	65.2 (61.9–66.2)	64.5 (63.9–65.5)	**0.0226**
BCMI	9 (7.6–11.3)	10.6 (8.3–12.3)	10.1 (5.1–12.7)	10.3 (6.0–12.8)	**0.0313**
BMR (Kcal)	1492.2 (1220.7–1730.4)	1532 (1291.8–1841.5)	1551 (1058–1753)	1619 (1220–1810)	ns
QRFT 1/2 left (cm)	1.63 (0.85–2.15)	1.86 (1.46–2.52)	1.27 (1.34–1.87)	1.34 (1.2–1.84)	**0.0172**
QRFT 1/2 right (cm)	1.7 (1.06–196)	1.8 (1.46–2.18)	1.65 (1.15–1.7)	1.72 (1.15–1.92)	**0.0399**
QRFT 2/3 left (cm)	1.46 (0.96–2.16)	1.76 (1.25–2.1)	1.36 (0.84–1.46)	1.43 (1.00–1.53)	**0.0020**
QRFT 2/3 right (cm)	1.53 (1.02–1.83)	1.81 (1.28–2.24)	1.57 (0.87–1.67)	1.47 (0.82–1.67)	**0.0125**

The data are reported as medians (minimum–maximum). Abbreviations: BMI, body mass index; BCM, body cell mass; ECW, extracellular water; FFM, fat free mass; FM, fat mass; TBW, total body water; BCMI, body cell mass index; BMR, basal metabolism; QRFT, quadriceps rectus femoris thickness; n.s., not significant; T0, enrollment time; T1, after twelve weeks.

**Table 3:** Handgrip strength test (HGST) of the plant-based bars (PBBs) group and of the control group performed at the two time points during this study.

	PBBs Group		Control Group		*p*-Value
Parameter	T0	T1	T0	T1	
HGST right	29.6 (8.7–39.8)	30.1 (5.5–37.3)	23.6 (23.6–23.9)	22.7 (21.7–25.3)	ns
HGST left	28.5 (13.3–43.1)	28 (10.4–39.5)	26.5 (18.9–27.5)	23.8 (19.1–24.8)	ns

The data are reported as medians (minimum–maximum). Abbreviations: HGST, handgrip strength test; n.s., not significant; T0, enrollment time; T1, after twelve weeks.

**Table 4:** Systolic and diastolic blood pressures of the plant-based bars (PBBs) group and of the control group measured at the two time points during this study.

	PBBs Group		Control Group		*p*-Value
Parameter	T0	T1	T0	T1	
Systolic BP (mmHg)	135 (100–160)	130 ± 15.3	140 (110–145)	144 (105–148)	ns
Diastolic BP (mmHg)	78.5 (60–97)	73 ± 10.6	72 (69–80)	70 (71–75)	ns

The data are reported as mean ± standard deviation for the parameters with normal distribution and as medians (minimum–maximum) for the non-normal variables. Abbreviations: BP, blood pressure; n.s., not significant; T0, enrollment time; T1, after twelve weeks.

**Table 5:** Composition of plant-based bars tested.

Name	Ingredients (%)	TAC mg GAE/bar	AA %	Kcal/bar
Green bar	dates 31.97%, cashews 14.04%, grapes 12.93%, raw cocoa butter 8.61%, figs 8.27%, extra virgin olive oil 5.72%, micronized grape pomace 2.8%, apple 2.59%, carob 2.5%, fennel 1.72%, cabbage 1,72%, spinach 1.72%, barley grass 1.2%, micronized grape seeds 1.4%, olive leaf powder 1.22%, lemon flavor 0.77%, kiwi 0.21%, salt 0.09%, saffron 0.1%	164.9	82.8	12
Blue bar	dates 32.28%, grapes 18.08%, cashews 10.33%, raw cocoa butter 8.6%, extra virgin olive oil 5.71%, prune 4.3%, micronized grape pomace 2.8%, carob 2.5%, acerola 1.71%, cabbage 1.71%, beet 1.71%, micronized grape seeds 1.4%, olive leaf powder 1.31%, acai 0.86%, blueberry 0.96%, rhubarb 0.43% kiwi 0.21%	205	85.7	129

Abbreviations: AA, anti-radical activity; GAE, gallic acid; TAC, total antioxidant activity.

**Table 6:** Nutritional information of plant-based bars tested.

	Green Bar 100 g	Blue Bar 100 g
Fat	18.2 g	19 g
of which saturated	8 g	7.7 g
Carbohydrate	47.5 g	49.9 g
of which sugars	44.9	45.4 g
Fiber	6.3 g	6.7 g
Protein	6.1 g	5.7 g
Salt	0.2 g	0.1 g

## Data Availability

The datasets presented in this article are not readily available because it contains personal patients’ data. Requests to access the datasets should be directed to the corresponding authors.
